# Congenital lobar emphysema in an adult

**DOI:** 10.4103/0970-2113.76307

**Published:** 2011

**Authors:** Mir Sadaqat, Javaid A. Malik, Raiesa Karim

**Affiliations:** *Departments of Internal Medicine, Chest Medicine, Sher-i-Kashmir Institute of Medical Sciences, Srinagar, India*; 1 *Departments of Internal Medicine, Medical College, Srinagar, India*

**Keywords:** Adult male, CLE, exertional breathlessness

## Abstract

Congenital lobar emphysema (CLE) is a clinico-radiological diagnosis, seen usually by four-six weeks of age (50% of patients) and rarely (<5% of patients) after the age of six months. Here, we report a young male with gradual onset of mild exertional breathlessness and physical examination revealing the features of right sided pneumothorax. X-ray of chest, with subsequent CT of chest, leads to the diagnosis of CLE. The pulmonary function tests, bronchoscopic examination and α_1_-antitrypsin level are normal. Patient is managed conservatively.

## INTRODUCTION

Developmental lung anomalies in the adult can be a challenge, as the abnormality may be mistaken for something more sinister. Congenital lobar emphysema (CLE) is a lung bud (bronchopulmonary) anomaly, usually detected in the neonatal period and early childhood. CLE is almost always unilateral with a male preponderance (M: F=3:1). This disease is characterized by hyperinflation of one or more lobes of the lung, leading to compression atelectasis on the ipsilateral or contralateral side and mediastinal shift. These, in turn, produce ventilation- perfusion mismatch.[1] We report this rare case from a northern state of India with the peculiarity of being found in an adult who is on conservative treatment.

## CASE REPORT

**Figure 1:**
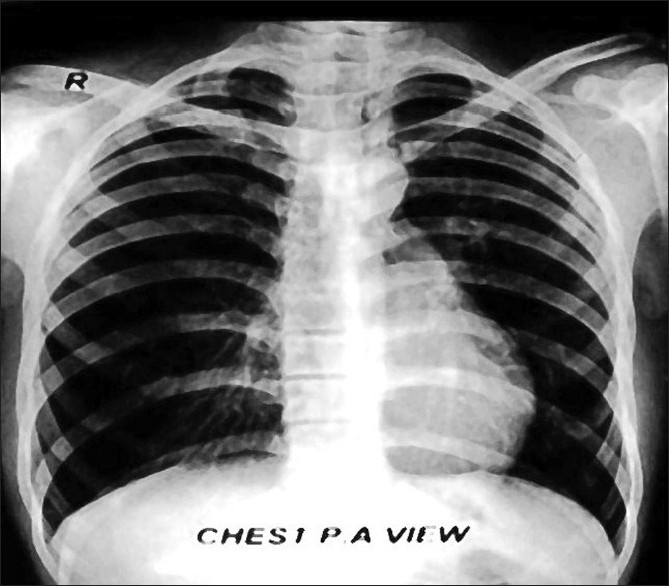
Chest X-ray showing mediastinal shift to left, crowded vascular markings along right paracardiac area and attenuated vascular markings in upper and mid zones of right lung

A 19-years-old male presented to our hospital with mild breathlessness on exertion from two months, denying any history of fever, hemoptysis or cough. On general physical examination, pulse of 82 beats per minute, blood pressure of 120/70 mmHg and respiratory rate of 16 per minute were recorded. The trachea was shifted toward left side. Examination of chest revealed a bulge in the right- posterior axillary area, leading to a clear asymmetry of the chest. Percussion note over the bulged area was hyper-resonant as compared to the opposite side. Tactile vocal fremitus and breath sounds were decreased in intensity over the abnormal area. Cardiac and abdominal examination was normal, except cardiac apex being 2 cm lateral to the midclavicular line. Immediately X-ray of chest was done, which showed tracheal shift to left, hyperlucent right lung, crowded vascular markings toward right paracardiac area, attenuated but maintained vascularity in the hyperlucent lung and notably no pleural demarcation of pneumothorax [Figure 1]. Arterial blood gas analysis of the patient showed pO_2_ -86 mmHg, saturation -94%, pCO_2_ -42 mmHg and pH -7.39. Serum potassium was 4.1 meq/l, sodium 136 meq/l and bicarbonate 25 meq/l. Electrocardiography and blood chemistry was normal. CT of chest featured as hyperexpansion of the posterior segment of right upper lobe with attenuated vascular markings and compression of right middle lobe and right lower lobe. The mediastinum was seen shifted toward left side but no air in pleural cavity was noted. The posterior segment of right upper lobe bronchus was seen traversing the hyperlucent lung parenchyma with no pleural demarcation of pneumothorax [Figures 2a and b]. No bronchocele or intraluminal foreign body was seen and lung parenchyma was normal. Left lung was free of any abnormality. In addition, no intrinsic or extrinsic mass lesion of the airways was identified. Pulmonary function tests were FEV_1-_86.66% of predicted, FVC-90.60% of predicted, FEF _25-75_ -77.96% of predicted, VC -2.48L, FEV1/VC-88.3% and MVV -73.6L. Serum α_1_ -antitrypsin level was 121 mg/dl. Then the patient was subjected to bronchoscopic examination to find any intraluminal etiology, but revealed no abnormality. Lastly, echocardiography was done to find any cardiac lesion that was normal.

**Figure 2a:**
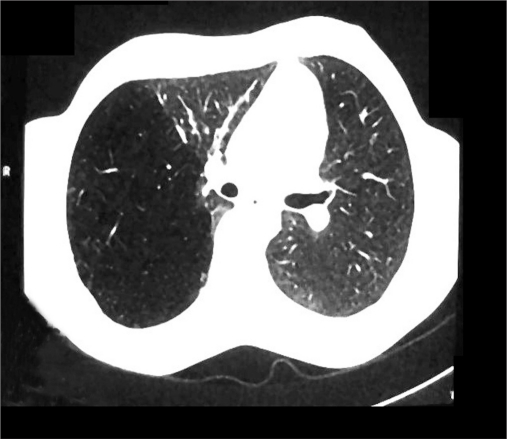
CT of chest showing hyperexpansion of posterior segment of right upper lobe and attenuated vascular markings

**Figure 2b:**
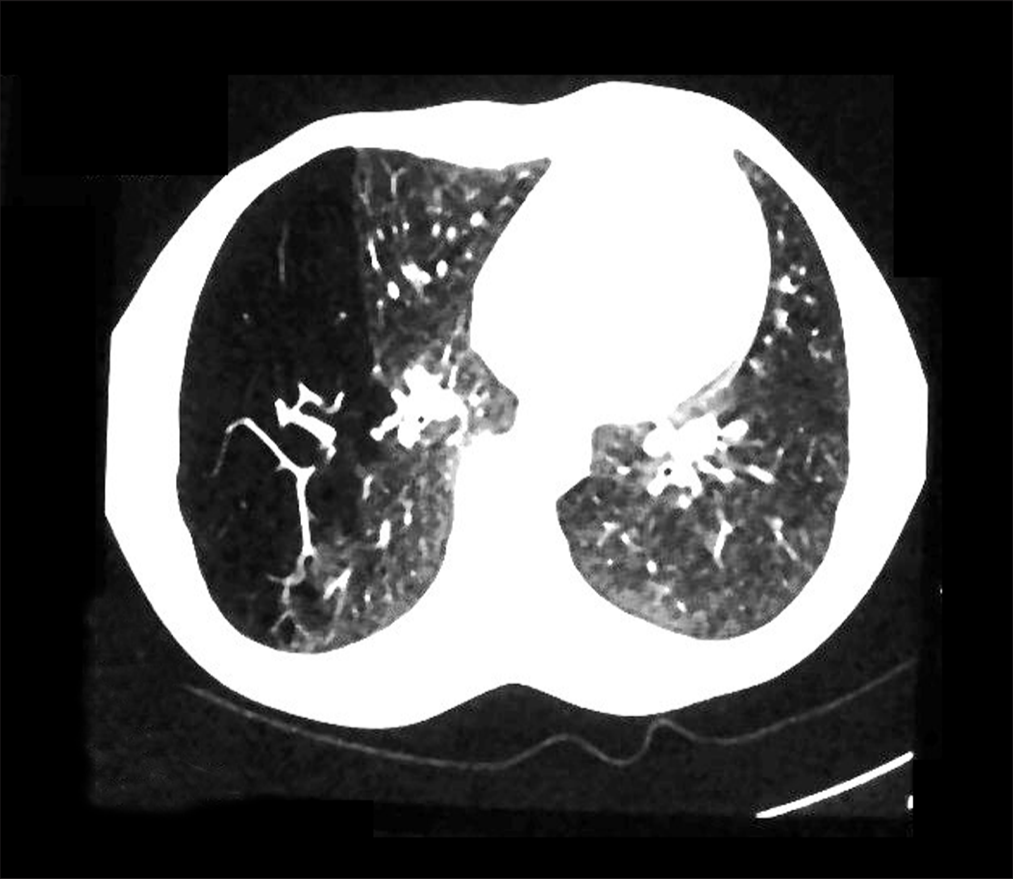
CT of chest showing right upper lobe bronchus traversing the hyperlucent lung parenchyma

Congenital lobar emphysema (CLE) was the only possibility in this patient because clinical and radiological features were absolute for this rare diagnosis. Patient is in our close follow up and is on conservative treatment because the symptoms were mild and he declined surgical option. Till last follow-up visit, patient was stable with no clinical progression of the disease based on frequent clinical assessments with serial X-rays of chest, pulmonary function tests and arterial blood gas analysis.

## DISCUSSION

CLE is characterized by variable severe overinflation of a pulmonary lobe, and compression of surrounding lung parenchyma. Pulmonary vascular markings can be seen up to the periphery of the affected lobe and there is no pleural demarcation as we see in pneumothorax. Mostly single lobe on one side is involved; however, multilobar involvement can be seen. Pulmonary lobes commonly involved are left upperlobe (43%), right middle lobe (32%), and right upper lobe (20%).[1] Adult presentation is unusual. They usually come with exertional dyspnoea or pulmonary infections. Symptoms can be mild and sometimes no symptoms can be seen.

The etiology of CLE is difficult to determine and no apparent cause is found in 50% of cases. Most commonly, the cause is a congenital defect of cartilage, ranging from hypoplastic and flaccid tissue to complete absence, accounting for 25% of cases. The remaining 25% have other causes of bronchial obstruction, such as redundant mucosal fold, mucus plugging, anomalous cardiopulmonary architectures and rarely intrathoracic masses.[2]

There are now data from molecular, genetic and embryonic organ culture studies that indicate that normal primary branching pattern of lung development is regulated by reiterative signaling of a fibroblast growth factor-10 pathway. Secondary branching appears to be dependent on the influence of sonic hedgehog and one of the Homeobox genes, Nkx 2.1, also identified as thyroid transcription factor-1. Although significant mutations in the genetic material controlling these functions appear to result in major anomalies, minor errors in transcription might result in localized deficiencies in the bronchial cartilage leading to the development of CLE. The reiterative nature of signaling also might help in explaining cases in which multiple lobes are affected.[3]

Association between congenital heart disease and CLE has been described. Murray *et al*. showed a 14% incidence of congenital heart diseases among 166 CLE patients. Common cardiac defects were left to right shunt, tetrology of fallot, right sided aortic arch and patent ductus arteriosus.[4,5]

Pulmonary function tests in CLE can be normal in asymptomatic or mild disease, but obstructive pattern is a feature of moderate to severe disease. Radiologically, the cardinal features are overinflation and air trapping, the former is manifested by markedly increased volume of the affected lobe, depressing the ipsilateral diaphragm, compressing the surrounding lung parenchyma and displacing the mediastinum. Vascular markings in the affected lobe are attenuated but maintained till the periphery of the lung. There is no line of pleural demarcation in contrast to pneumothorax. Chest X-ray and CT scan usually establish the diagnosis. Other imaging modalities include MRI and V/Q scan. MRI demonstrates any vascular lesion causing external compression but it is only an adjunct to the diagnosis of CLE, not a primary investigation. V/Q scan depicts ventilation defects and decreased perfusion in the hyoperexpanded lobe due to attenuated vascularity.[6]

Pulmonary lobectomy of involved lobe is the treatment of choice for CLE.[7] PS Critchley and colleagues[8] reported a young pregnant female with left upper lobe CLE and managed by the resection of the involved lobe.[8] Granato F[9] reported first case of endoscopic parenchymal sparing resection in CLE with mild symptoms. Thus video assisted thoracoscopic surgery (VATS) seems to be an emerging and advantageous approach. Dutta *et al*.[10] from India reported conservative treatment of a neonate with CLE and Meir Mei-Zahav *et al*.[11] managed 14 children (0-17 years) conservatively out of 20 children with CLE. However, studies are further required to determine the long-term outcome of conservative management, as the disease is rare in adults. Going through the literature, surgical option is the suitable one and VATS can be promising in future.
